# Effective alcohol policies are associated with reduced consumption among demographic groups who drink heavily

**DOI:** 10.1111/acer.15030

**Published:** 2023-04-23

**Authors:** Sally Casswell, Taisia Huckle, Karl Parker, Thomas Graydon‐Guy, June Leung, Charles Parry, Perihan Torun, Gantuya Sengee, Cuong Pham, Gaile Gray‐Phillip, Sarah Callinan, Surasak Chaiyasong, Anne Marie MacKintosh, Petra Meier, Steve Randerson

**Affiliations:** ^1^ SHORE & Whariki Research Centre College of Health, Massey University Auckland New Zealand; ^2^ Alcohol, Tobacco and Other Drug Research Unit, South African Medical Research Council Cape Town South Africa; ^3^ Department of Public Health Hamidiye International Medical School Istanbul Turkey; ^4^ Public Health Policy and Coordination Department National Center for Public Health of Mongolia Ulaanbaatar Mongolia; ^5^ Center for Injury Policy and Prevention Research (CIPPR) Hanoi University of Public Health Hanoi Vietnam; ^6^ National Council on Drug Abuse Prevention St Kitts and Nevis; ^7^ Centre for Alcohol Policy Research (CAPR), School of Psychology and Public Health La Trobe University Melbourne Victoria Australia; ^8^ International Health Policy Program (IHPP), Ministry of Public Health & Faculty of Pharmacy Mahasarakham University Maha Sarakham Thailand; ^9^ Institute for Social Marketing and Health, Faculty of Health Sciences and Sport University of Stirling Stirling UK; ^10^ School of Health and Related Research University of Sheffield Sheffield UK; ^11^ Present address: MRC/CSO Social and Public Health Sciences Unit University of Glasgow Glasgow UK

**Keywords:** alcohol policy index, education, heavy drinking, high‐middle‐income countries, young people

## Abstract

**Background:**

Alcohol policies stand out among other noncommunicable disease‐relevant policies for the lack of uptake. Composite indicators have been developed to measure the effects of alcohol control policy. We investigated whether drinking patterns among demographic groups from general population samples of drinkers from diverse countries are associated with alcohol control policy as measured by the International Alcohol Control (IAC) Policy Index.

**Methods:**

Representative samples of adult drinkers from 10 countries (five high‐income and five middle‐income) were surveyed about alcohol consumption, using beverage and location‐specific questions.

**Measurements:**

The IAC Policy Index was analyzed with frequency, typical occasion quantity, and volume consumed. Analyses used mixed models that included interactions between country IAC Policy Index score and age group, gender, and education level.

**Findings:**

Each increase in IAC policy index score (reflecting more effective alcohol policy) was associated with a 13.9% decrease in drinking frequency (*p* = 0.006) and a 16.5% decrease in volume (*p* = 0.001). With each increase in IAC Policy Index score, both genders decreased for all three measures, but men less so than women. Women decreased their typical occasion quantity by 1.2% (*p* = 0.006), frequency by 3.1% (*p* < 0.001), and total volume by 4.2% (*p* < 0.001) compared to men. Low and mid‐education groups decreased their typical occasion quantity by 2.6% (*p* < 0.001) and 1.6% (*p* = 0.001), respectively, compared to high education, while for drinking frequency the low education group increased by 7.0% (*p* < 0.001). There was an overall effect of age (*F* = 19.27, *p* < 0.0001), with 18–19 and 20–24‐year‐olds showing the largest decreases in typical occasion quantity with increasing IAC policy index score.

**Conclusions:**

The IAC Policy Index, reflecting four effective policies, was associated with volume and frequency of drinking across 10 diverse countries. Each increase in the IAC Policy Index was associated with lower typical quantities consumed among groups reporting heavy drinking: young adults and less well‐educated. There is value in implementing such alcohol policies and a need to accelerate their uptake globally.

## Graphical abstract

In 10 diverse countries the IAC Policy Index, which measures the strength of four effective alcohol policies, was found to be associated with the volume of alcohol consumed and the frequency of drinking among their populations. Younger people, who are heavier drinkers, showed the largest decrease in typical occasion quantity as the IAC Policy Index score indicated stronger alcohol control measures and those with low education decreased their consumption to a greater extent relative to those with more years of education.

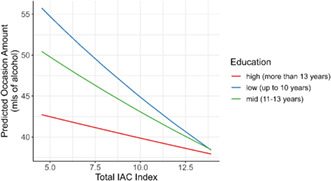

## INTRODUCTION

### Global context

In 2018, alcohol‐attributable deaths amounted to 3 million globally per annum. Alcohol is a risk factor for noncommunicable diseases which account for the majority of premature deaths worldwide and also contribute to the severity of reaction to COVID‐19. Alcohol also makes a major contribution to the health burden via its causal role in injury (Chikritzhs & Livingston, 2021). Alcohol‐attributable deaths and disability are experienced among younger groups in the population, and approximately half of alcohol‐attributable deaths occur before the age of 60 (Shield et al., 2020).

The global burden from alcohol is expected to rise due to increased consumption in low‐ and middle‐income countries (LMICs), especially in South East Asia and the Western Pacific regions of the world, if effective policies requiring regulation of supply and marketing and increased taxation are not implemented (Manthey et al., 2019). The alcohol‐attributable burden is higher in countries with low Human Development Index scores (Shield et al., 2020) making a policy response an issue of health equity.

Alcohol policies stand out among other noncommunicable disease‐relevant policies for the lack of uptake (Allen et al., 2020). In 2020, the Executive Board of the World Health Assembly, in response to concern expressed largely by LMICs (Casswell & Rehm, 2020), requested the Director General to develop an action plan for the global strategy to reduce the harmful use of alcohol and to adequately resource work on the harmful use of alcohol (World Health Organization, 2020). A collaboration between UN agencies and civil society has developed a package to promote effective alcohol policies, SAFER (World Health Organization, 2018). This highlights the need to develop effective tools to monitor uptake of effective alcohol policies at country level (Flor & Gakidou, 2020).

### Alcohol policy indices

A number of composite indicators have been developed to measure effects of alcohol control policy. Most studies have looked for a relationship of indices with per capita alcohol consumption (APC; Brand et al., 2007; Ferreira‐Borges et al., 2015; Flor & Gakidou, 2020; Hadland et al., 2015; Karlsson et al., 2012) and several in high‐income countries (HICs) have examined the relationship with patterns of drinking (Leal‐López et al., 2020; Paschall et al., 2009) and alcohol harm (Hadland et al., 2015; Lira et al., 2020; Naimi et al., 2014).

The International Alcohol Control (IAC) study developed a policy index which used four policies established in a large body of research to be likely to be the most effective (and cost‐effective) in a range of settings (Babor et al., 2022; Chisholm et al., 2018). While some indices have used the existence of policies weighted by effectiveness (e.g., Brand et al., 2007) and some with the addition of implementation (e.g., Carragher et al., 2014), an innovation in the IAC Policy Index was the inclusion of measures of the way in which key aspects of the alcohol environment reflected the intention of the policies (policy impact) such as the actual hours at which alcohol was available for sale, affordability of alcohol products, and the extent to which drink driving legislation was enforced (Casswell et al., 2022).

The IAC Policy Index was strongly associated with recorded APC in a number of diverse country settings and showed a larger relationship than previously published indices. Affordability and marketing of alcohol were especially highly correlated with APC (Casswell et al., 2022).

### Alcohol indices and drinking patterns

Studies which have examined either alcohol policy indices or a range of policies in relation to drinking patterns have typically focused on specific sectors of the population: adolescents (Paschall et al., 2009), young males (Foster et al., 2019) mid to older age groups (Sandoval et al., 2019). Cross‐sectional analysis in HICs has found heavier drinking associated with less restrictive policy in young males (Foster et al., 2019) and more frequent drinking among adolescents was associated with less restrictive policy (Paschall et al., 2009). Longitudinal analysis of adolescent drinking in 33 largely HIC and regions over 12 years found a combination of alcohol control policies was more effective in reducing adolescent drinking outcomes than single policy measures. Reducing the affordability of alcohol stood out as the most successful single measure (Leal‐López et al., 2020). Policy changes over time were not found to reduce the relationship between heavy drinking and lower educational status in mid to older adults (Sandoval et al., 2019).

This is one of the first analyses to examine the relationship between a Policy Index and drinking patterns for demographic groups among general population samples of drinkers from diverse countries, including HIC and LMIC (International Alcohol Control Policy Evaluation Study, 2018). The aim of the paper is to investigate whether the IAC Policy index is associated with drinking patterns within the population overall and the different demographic groups including by age, gender, and educational status.

## METHODS

### Design

Cross‐sectional analyses of alcohol policy stringency and impact ratings based on the International Alcohol Control Policy Index and drinking patterns in 10 high‐ and middle‐income countries: Australia, New Zealand, England, Scotland, South Africa, Mongolia, Thailand, Vietnam, Saint Kitts, Nevis, and Turkey. This was a small but diverse sample of countries, based on researchers who obtained funding and were willing to participate.

### IAC survey data

Sampling methods were designed to obtain a random representative sample of adult drinkers aged 16–65 years and each country utilized the sampling frame that was most appropriate in their context (face‐to‐face or telephone sample frames were used; Table 1).

**TABLE 1:** Summary of data collection methods for IAC countries.

Country	Survey year	Age range	Sampling scope	Survey mode	Response rate (%)
Australia	2013	16+	National	Telephone/mobile	37
England	2012/13	16–65	National	Telephone	16
Scotland	2012/13	16–65	National	Telephone	19
New Zealand	2011	16–65	National	Telephone	60
St Kitts & Nevis	2014/16	16–65	National	Face to face	60
Thailand	2012/2013	15–65	National	Face to face	93
South Africa	2014	16–65	Tshwane metropolitan municipality (covering Pretoria)	Face to face	78
Peru	2015	16–65	Los Olivos District, City of Lima	Face to face	82
Mongolia	2013	16–65	Ulaanbaatar (2 districts)	Face to face	44
Viet Nam	2014	16–65	Three provinces (Thai Binh, Khanh Hoa, and Dong Thap)	Face to face	99

Multi‐stage sampling of geographical units was used to represent St Kitts and Nevis, Thailand; Tshwane metropolitan municipality (covering Pretoria) in South Africa; two districts in Ulaanbaatar (Bayanzurkh and Chingeltei) in Mongolia; and three provinces in Vietnam (Thai Binh, Khanh Hoa, and Dong Thap) and Istanbul in Turkey. In New Zealand, a national stratified sample of residential landline numbers comprised the sample frame, including published and unpublished landline numbers. Scotland and England conducted a stratified sample utilizing the same approach. In Australia, a national sample frame of residential landline and cell phone numbers was used (60% residential landline and 40% cell phone numbers). Once a household was recognized as residential, numerous callbacks were made at different times of the day and days of the week in order to attempt to reach the household. Once a household was contacted, eligible individuals were enumerated, and one respondent was selected at random by the computer/tablet. A screening interview established eligibility for participation in the study (drinking in the past 6 months and age 16–65 years). The oversample of risky drinkers obtained in Australia was accounted for in all analyses with weighting. Surveys were collected between 2011 and 2016. For detailed description of the methods, see Huckle, Casswell, et al. (2018). The median response rate for all countries was 60% (range 16%–99%). Response rates were calculated using at least American Association for Public Opinion Research formula #3 (American Association for Public Opinion Research, 2015).

The survey utilized a within‐location beverage‐specific framework (Casswell et al., 2002; Huckle, Casswell, et al., 2018). Countries adapted this consumption measurement framework to their country context in terms of locations and beverages including unrecorded (untaxed/informal) as well as commercial alcohol (Huckle, Casswell, et al., 2018). Respondents reported their consumption of the different beverages relevant to their country in their own terms and interviewers coded these by using containers and glass sizes in which alcohol is commonly served and sold in that country (Huckle, Casswell, et al., 2018). In this way, respondents did not have to “calculate” and report their consumption in terms of standard drinks which is likely to introduce error (World Health Organization, 2000). Calculation of the quantity of mL of EtOH was made based on alcohol content for each beverage and container sizes in each country. Each country's data were converted to mls of EtOH based on alcohol content and container sizes, and location and beverage‐specific data were transformed into summary consumption variables which were comparable across countries (Huckle, Casswell, et al., 2018).

This survey methodology provides comparable and robust survey data on drinking patterns (Casswell et al., 2012, 2014, 2016). Where data on alcohol available for consumption were available, the validity of survey consumption measures was assessed by calculating survey coverage and was found to be 86% or above (Huckle, Casswell, et al., 2018). The high level of coverage reflects that respondents are not required to “calculate” standard drinks to report their consumption, asking by location likely helps respondents to remember their drinking occasions and the variation in drinking patterns captured in different locations, that is, the measure captures the reality that drinking occasions are typically heavier in some locations than others (Huckle, Casswell, et al., 2018). Nondrinkers were not included in the survey as the focus was on policy influences shaping drinking patterns. A separate paper has examined the relationship of the IAC Policy Index on abstention as defined by GISAH (Leung et al., 2022).

In addition to the strength of the survey measures and the diverse countries (high‐income and middle‐income) included, the combined sample of drinkers is large and allows for detailed investigation of the policy index in different demographic groups.

### Measures

#### IAC policy index

The IAC Policy Index score was developed based on the most effective alcohol policies, three “best buys” (restrictions on availability, marketing, and pricing policies) and one “good buy” (drink‐driving prevention) as determined by effectiveness research, and was innovative in its inclusion of impact measures (reflecting implementation as well as policy stringency). Details of its development and calculation can be found in Casswell et al., 2022 (Casswell et al., 2022). The Index was validated against APC in 12 countries (Casswell et al., 2022). The index score across the ten countries ranged from 5.0 to 13.9 with a higher score indicating more stringency and more impact of alcohol policy in the alcohol environment (Casswell et al., 2022).

#### Typical occasion quantity, frequency, and volume

Drinkers in the past 6 months were asked how often they drank and how much they would drink on a typical occasion at a number of mutually exclusive specified locations, plus any additional locations, where they had had a drink (Huckle et al., 2012). For a list of country‐specific locations, please see (Gray‐Phillip et al., 2018; Huckle et al., 2020). Each country's location and beverage‐specific data were transformed, using standardized procedures, into the summary consumption variables below.

#### Typical occasion quantity

Typical occasion quantity for an individual is the weighted average of all the typical occasion quantities reported by a respondent at each location, taking into account how often the respondent drank at the location in the past 6 months (mls of EtOH). In this way, a location that a person drank at once a year had minimal influence compared to a location drank at more regularly.

##### Annual frequency of drinking

The sum of all frequencies at all of the locations in the past 6 months.

##### Total volume in the past six months

Calculated by determining the volume of each beverage consumed (typical occasion quantity*frequency) and then summing across all locations (mls of EtOH).

#### Demographics

Demographic variables included were gender, age, and education. Gender was categorized as male or female. Age was categorized into groups: 16–17, 18–19, 20–24, 25–34, 35–44, 45–54, and 55–65 years. Education was categorized as low (up to 10 years of education), middle (10–13 years of education), or high (more than 13 years). Years of education has been used in this analysis as it has been shown to be a reliable cross‐country indicator of social status (Huckle, Romeo, et al., 2018).

#### Weighting

As one person was selected per household, unequal probability of respondent selection was corrected for. Australian survey weights accounted for the over‐sampling of risky drinkers.

### Analysis

The IAC Policy Index score was merged with the IAC survey data on drinking patterns to test the relationship between the alcohol consumption in a country and the index score it was given.

Outliers were removed by using an approach suitable for cross‐country complexities (described in detail elsewhere; Huckle, Casswell, et al., 2018). First, we transformed the typically right‐skewed distributions of consumption‐related variables to normalize them. The transforming function was logarithmic for typical occasion quantity and power function for frequency of drinking. Second, the transformed series was then centered and scaled by subtracting the mean and dividing by the standard deviation (the 99th percentile of respondents was then removed; Huckle, Casswell, et al., 2018).

Statistical analysis was performed using mixed models with country modeled using random intercepts. This was to address the nested structure of individuals within countries. Age group, gender, and education level were fitted as fixed effects and to control for the effects of these variables. In addition to the main effects, interactions between the three covariates and the IAC Policy Index score were explored individually to test for any differing effects between the index score and the different levels of age, gender, and education. Three consumption measures were used: typical occasion quantity, frequency, and total volume. All three variables were right skewed but residuals from the model were normally distributed after the responses were log‐transformed. There were 17,319 people in the total sample. Of these, 16,169 observations were included in the typical occasion quantity and total volume analysis, while 16,219 were included in the frequency model, due to missing values. All analyses were performed in R (version 4.1; R Core Team, 2021), using the lme4 package (Bates et al., 2015) for mixed models, and the ggplot2 package (Wickham, 2016) for figures.

## RESULTS

### Main effects

The average volume of alcohol consumed in the past 6 months was significantly associated with the IAC Policy Index. There was a 16.5% decrease in volume for each additional increase in IAC Alcohol Policy Index score which reflected more effective alcohol policies (*p* = 0.001). Frequency of drinking was significantly related to the IAC Policy Index with a 13.9% decrease in frequency (*p* = 0.006) for each additional increase in IAC Alcohol Policy Index score. The typical quantities consumed by respondents overall was not significantly related to the IAC Policy Index. (see Tables S1–S3).

A significant Interaction between gender and the IAC Policy Index score was found for all three consumption variables (Tables S1–S3). In each of the three measures, it was found that men's consumption decreased at a slower rate as the Index score increased, compared to women. With each increase in IAC Policy Index score, both genders decreased for all three measures but men did so less than women. This meant that women had a lower typical occasion by 1.2% (*p* = 0.006), frequency by 3.1% (*p* < 0.001), and total volume by 4.2% (*p* < 0.001) compared to men.

### Age group

**FIGURE 1:**
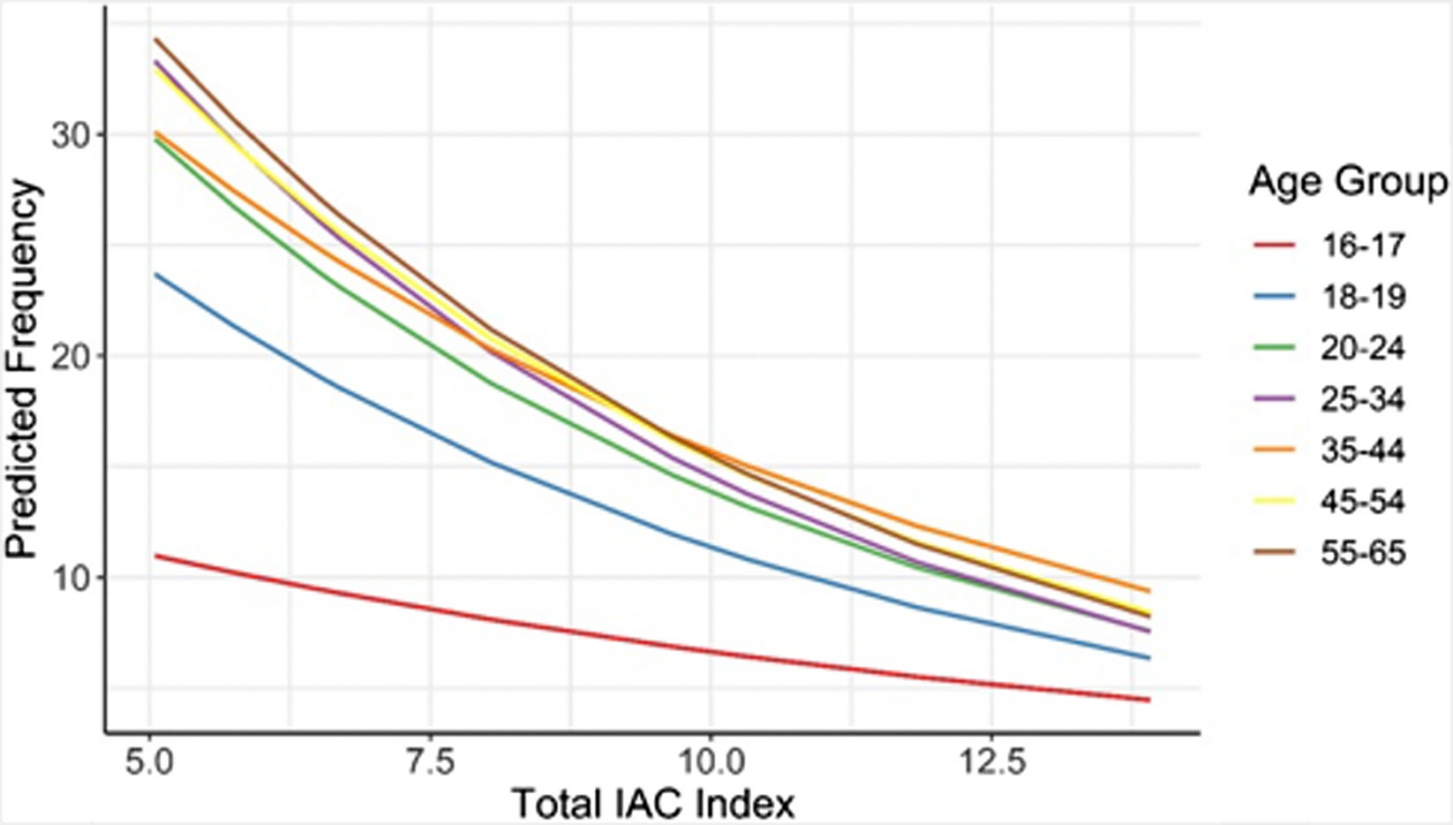
Interaction between IAC Policy Index score and age group for frequency of drinking.

Age group has a significant interaction with the IAC Policy Index score for the frequency model (*F* = 3.09, *p* = 0.005; see Table S1). The 16–17 age group had the lowest average frequencies over the range of IAC Policy Index scores. As the index score indicated stronger alcohol control measures, all age groups decreased relative to the 35–44 age group (Figure 1) but the age groups with the highest frequency decreased the most.

**FIGURE 2:**
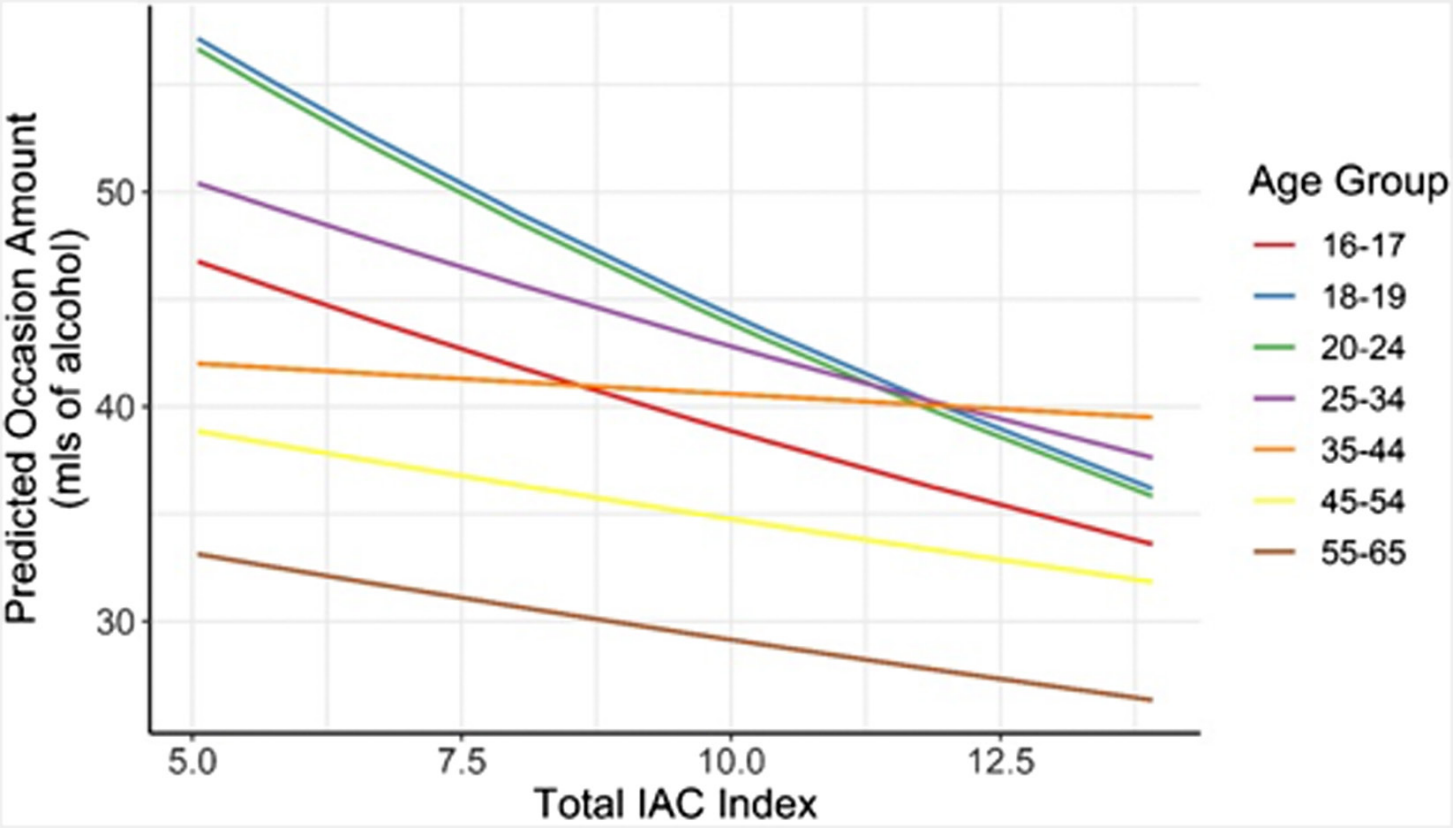
Interaction between IAC Policy Index score and age group for typical occasion quantity.

A significant interaction was found between age group and the IAC Policy Index score (*F* = 10.43, *p* < 0.001) for typical occasion quantity. The 18–19 and 20–24 age groups show the largest decrease in typical occasion quantity as the IAC Policy Index score indicated stronger alcohol control measures (Figure 2). The 35–44 age group shows little change in typical occasion quantity; when the index score indicates limited control measures, they are near the middle of the other age groups, remain relatively stable, and report the highest typical drinking occasion quantities when the IAC Policy Index score indicates stronger alcohol control measures (see Table S2).

### Education level

**FIGURE 3:**
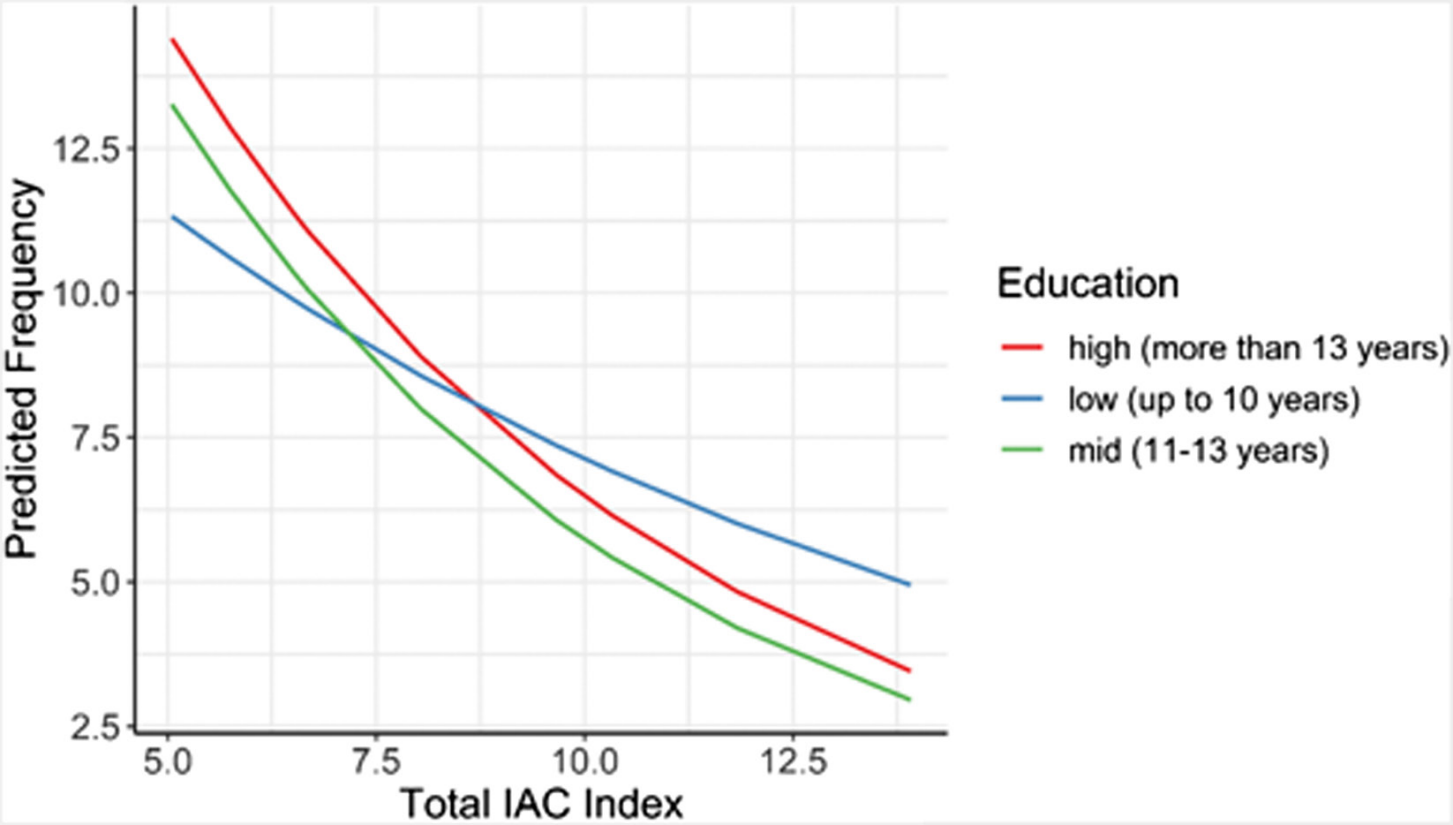
Interaction between IAC Policy Index score and education level for frequency of drinking.

Significant interactions were found between years of education and frequency of drinking (see Table S1). Frequency decreases with stronger alcohol control measures but those with fewer years of education decrease at a slower rate. As the IAC Policy Index score indicated stronger control measures, average frequency for the low education level was lower by 6.5% (*p* < 0.001) compared with high education levels (Figure 3).

**FIGURE 4:**
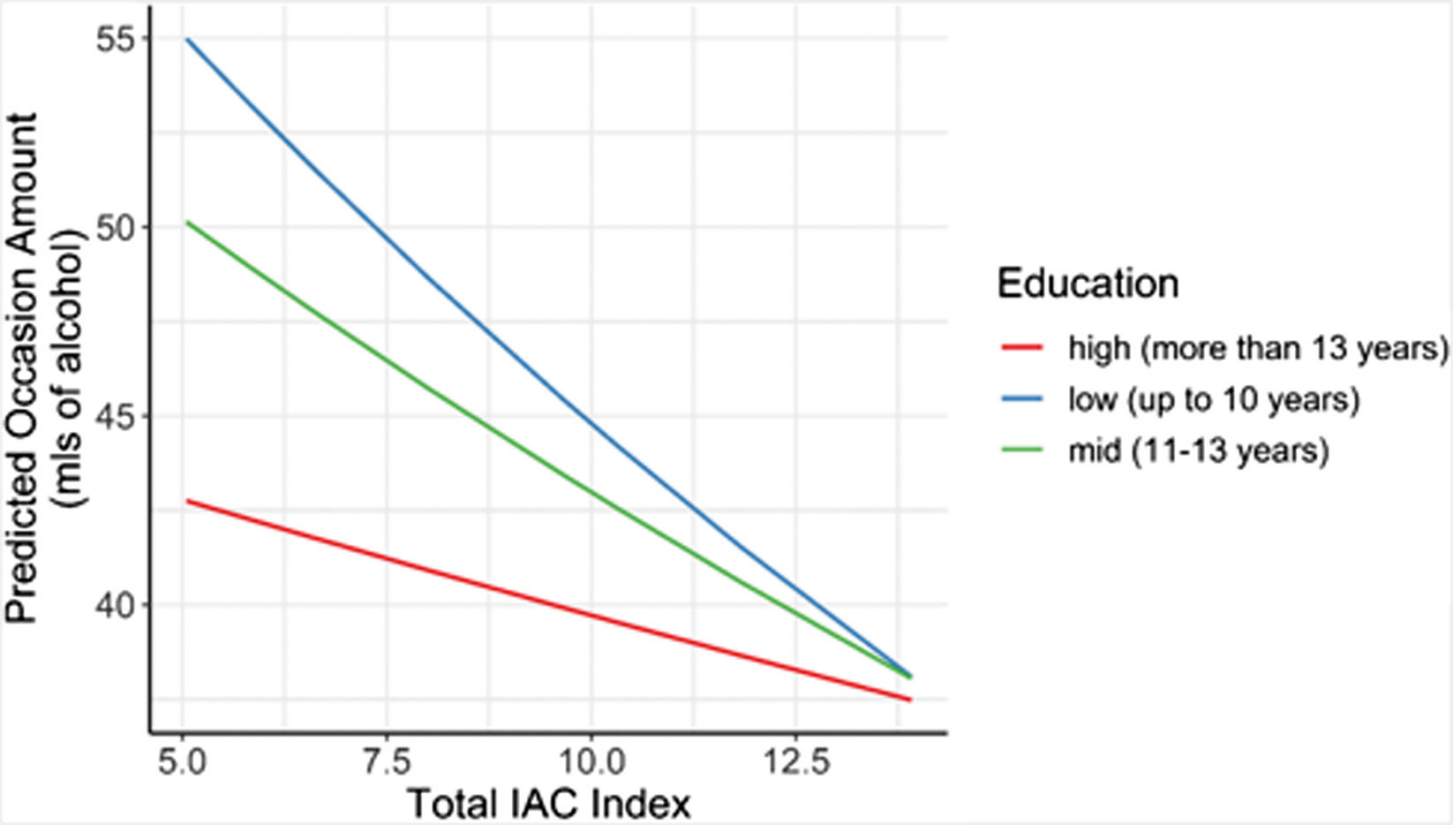
Interaction between IAC Policy Index score and education level for typical occasion quantity.

Fewer years of education is associated with the largest typical quantities consumed in a drinking occasion followed by mid and the high education level (see Table S2). However, when the IAC Policy Index indicates stronger alcohol control measures all three education levels converge to similar quantities consumed. Increases in control policy have a greater effect for those with fewer years of education than those with more (Figure 4), that is, low and mid‐education groups had a lower typical occasion quantity by 2.6%, (*p* < 0.001) and 1.6% (*p* = 0.001) respectively compared to high education.

## DISCUSSION

The average volume of alcohol consumed was significantly related to the IAC Policy Index. Volume is calculated from the reports of frequency and typical quantities consumed in drinking occasions using a location and beverage‐specific approach previously shown to have high validity as measured by coverage of alcohol available for consumption in HICs (Huckle, Casswell, et al., 2018).

Frequency of drinking and the quantities consumed tend to be correlated but nevertheless represent different dimensions of alcohol use. Frequency of drinking was significantly related to the IAC Policy Index overall in line with previous research which investigated the relationship between frequency of adolescent drinking and found an inverse relationship with policy effectiveness (Paschall et al., 2009). In the present analysis conducted among adults, as the strength of policies in place increased across countries, frequency of drinking decreased overall and among the demographic groups who drank most frequently relative to those who drank less frequently (e.g., older age groups and mid/high education).

Previous research has shown a strong inverse relationship between the strength of the policy environment of states in the U.S. and individual‐level binge drinking, and this significant relationship was observed consistently across age and gender (Xuan et al., 2015). In the present analysis of drinking patterns, the quantities per typical occasion typically consumed, while not showing a significant relationship overall, were significantly associated in the case of certain demographic groups: young adults and less well‐educated people.

The young adult groups, 18–19 and 20–24 years, showed the highest levels of typical quantities consumed at the lowest Index scores and, relative to older age groups, show a greater reduction in quantities consumed with more restrictive policy. Young adults experience disproportionate alcohol‐attributable deaths of which injury plays a large part (Rehm et al., 2006) and so the significant inverse relationship found between quantities consumed by this heavy drinking cohort and more restrictive alcohol policy supports the previous research indicative of an environment–behavior linkage of substantial public health significance.

The countries vary in education level according to the Education index of the Human Development Index (Casswell, 2018) and respondents' years of education interacted with the Index scores. As seen in previous research (Huckle et al., 2010), those in our study with lower educational status reported drinking larger typical quantities (Probst et al., 2020). This study is the first to find a stronger association between educational status and policy strength. The lower education group decreased their typical occasion quantity the most as policy strength increased. This meant in countries with stronger alcohol control policy those with lower educational status consumed similar quantities to the better educated. This has important public health implications given the contribution heavy episodic drinking makes to greater alcohol‐related mortality among lower socio‐economic sectors (Probst et al., 2020).

This suggests a very valuable impact of alcohol control policies in reducing high quantities consumed among some demographic groups in countries with relatively stricter alcohol policies. Larger quantities consumed are predictive of alcohol‐attributable injury and violence which make up a significant proportion of disability‐adjusted years of life lost (Shield et al., 2020) and are also predictive of negative impact on health and well‐being of close associates of the drinker (Casswell et al., 2011).

Men's drinking was less associated with the IAC Policy Index compared with women. There have been few gender analyses in evaluations of alcohol policy impacts with inconsistent findings (Fitzgerald et al., 2016), but some studies have suggested women are more sensitive to tax than men (Subbaraman et al., 2020).In recent analysis of response to minimum unit price (MUP), women have shown greater responsiveness (Connor, 2016; Dumont et al., 2017; Fitzgerald et al., 2016; Subbaraman et al., 2020) and this has been supported by modeling of the impact of MUP (Meier et al., 2021). However, although the male respondents were less likely to decrease their consumption in relation to the policy index score relative to females, males in countries with strong alcohol policy had considerably lower volume, frequency, and typical quantities than males in countries with weaker polices in place.

### Limitations

This is a cross‐sectional study, and the associations between policy status and drinking patterns are susceptible to reverse causation. Future longitudinal analyses of drinking patterns over time may provide more clarity on the direction of relationships (Leung et al., 2022). In cultures such as Turkey where drinking is prohibited by religious traditions, these traditions may affect the habits of drinkers. Our measurements focused on the most effective alcohol policies but did not measure all policies. It is possible that the presence of other unmeasured policies could contribute to the effects size observed with these four policies. The study also used a small convenience sample of countries, and findings may not be representative or inferred to a broader sample of countries. However, the sample did include countries with varying levels of effective alcohol policies in place, from strong to weak, providing evidence of policy effectiveness in this sample. Response rates were high in many countries but were low in Australia, England, and Scotland, although the Australian response rate was in the normal range of response rates for telephone surveys in Australia (Callinan et al., 2016; Huckle, Casswell, et al., 2018). In England and Scotland, a high number of “no answers” and “no screener completed—unknown if eligible respondent” contributed to the lower response rates (Huckle, Casswell, et al., 2018). Surveys with low response rates will generally miss some heavy drinkers (Tolonen et al., 2019) and young people (Meiklejohn et al., 2012) and capture more relatively well‐educated respondents (Caetano, 2001). This could have underestimated typical quantities consumed and overestimated frequency of drinking in this study.

## CONCLUSION

The IAC Policy Index, reflecting a country's uptake of the most effective alcohol policies, was significantly inversely related with self‐reported volume and frequency of drinking across 10 diverse countries. Stronger alcohol control policy, as reflected in the IAC Policy Index, also showed a significant inverse relationship with lower levels of typical quantities consumed among demographic groups which report heavy drinking: young adults and those with fewer years of education. This suggests the value of implementing such alcohol policies and the need to accelerate their uptake globally.

## CONFLICT OF INTEREST STATEMENT

The authors have no conflicts on interest to declare.

## Supporting information


Tables S1–S3

Appendix S1
