# Supplementary material for: Effective alcohol policies are associated with reduced consumption among demographic groups who drink heavily
Source: Alcohol Clin Exp Res (Hoboken). 2023 Apr 23;47(4):786–95. doi: 10.1111/acer.15030 (PMC10947406; doi:10.1111/acer.15030)
Supplement: Supplementary file 1 — Tables S1–S3 [file ACER-47-786-s002.docx]

Table S1: Model estimates for main and interaction effects (education, gender and age group) for frequency of drinking

|  | **Main effects** | | | | | | | | | | | | | | | **Education Interaction** | | **Gender Interaction** | | **Age Interaction** | |
| --- | --- | --- | --- | --- | --- | --- | --- | --- | --- | --- | --- | --- | --- | --- | --- | --- | --- | --- | --- | --- | --- |
| *Predictors* | *Estimates* | | | | | | | | | | | | | | *p* | *Estimates* | *p* | *Estimates* | *p* | *Estimates* | *p* |
| (Intercept) | 3.39 | | | | | | | | | | | | | | **<0.001** | 3.48 | **<0.001** | 3.54 | **<0.001** | 2.91 | **<0.001** |
| Age 16-17 (ref) |  |  |  |  |  |  |  |  |  |  |  |  |  |  |  |  |  |  |  |  |  |
| Age 18-19 | 0.55 | | | | | | | | | | | | | | **<0.001** | 0.53 | **<0.001** | 0.56 | **<0.001** | 1.01 | **0.001** |
| Age 20-24 | 0.76 | | | | | | | | | | | | | | **<0.001** | 0.73 | **<0.001** | 0.77 | **<0.001** | 1.27 | **<0.001** |
| Age 25-34 | 0.81 | | | | | | | | | | | | | | **<0.001** | 0.79 | **<0.001** | 0.81 | **<0.001** | 1.44 | **<0.001** |
| Age 35-44 | 0.86 | | | | | | | | | | | | | | **<0.001** | 0.83 | **<0.001** | 0.86 | **<0.001** | 1.16 | **<0.001** |
| Age 45-54 | 0.86 | | | | | | | | | | | | | | **<0.001** | 0.84 | **<0.001** | 0.87 | **<0.001** | 1.37 | **<0.001** |
| Age 55-65 | 0.89 | | | | | | | | | | | | | | **<0.001** | 0.87 | **<0.001** | 0.89 | **<0.001** | 1.44 | **<0.001** |
| Education Low (up to 10 | -0.01 | | | | | | | | | | | | | | 0.795 | -0.58 | **<0.001** | -0.01 | 0.807 | -0.01 | 0.834 |
| years) |  |  |  |  |  |  |  |  |  |  |  |  |  |  |  |  |  |  |  |  |  |
| Education Mid (11-13 years) | -0.12 | | | | | | | | | | | | | | **<0.001** | -0.04 | 0.604 | -0.12 | **<0.001** | -0.12 | **<0.001** |
| Education High (13+ years) (ref) | | | | | | |  |  |  |  |  |  |  |  |  |  |  |  |  |  |  |
| Gender [Male] | 0.71 | | | | | | | | | | | | | | **<0.001** | 0.71 | **<0.001** | 0.45 | **<0.001** | 0.71 | **<0.001** |
| Gender [Female] (ref) | | |  |  |  |  |  |  |  |  |  |  |  |  |  |  |  |  |  |  |  |
| IAC Index | -0.15 | | | | | | | | | | | | | | **0.006** | -0.16 | **0.003** | -0.17 | **0.002** | -0.1 | 0.092 |
| Education Low (up to 10 |  | | | | | | | | | | | | | |  | 0.07 | **<0.001** |  |  |  |  |
| years) * IAC Index |  |  |  |  |  |  |  |  |  |  |  |  |  |  |  |  |  |  |  |  |  |
| Education Mid (11-13 years) * IAC Index |  | | | | | | | | | | | | | |  | -0.01 | 0.327 |  |  |  |  |
| Gender [Male] * IAC Index |  | | | | | | | | | | | | | |  |  |  | 0.03 | **<0.001** |  |  |
| Age 18-19* IAC Index |  | | | | | | | | | | | | | |  |  |  |  |  | -0.05 | 0.104 |
| Age 20-24* IAC Index |  | | | | | | | | | | | | | |  |  |  |  |  | -0.05 | **0.044** |
| Age 25-34* IAC Index |  | | | | | | | | | | | | | |  |  |  |  |  | -0.07 | **0.009** |
| Age 35-44* IAC Index |  | | | | | | | | | | | | | |  |  |  |  |  | -0.03 | 0.232 |
| Age 45-54* IAC Index |  | | | | | | | | | | | | | |  |  |  |  |  | -0.05 | **0.043** |
| Age 55-65* IAC Index |  | | | | | | | | | | | | | |  |  |  |  |  | -0.06 | **0.027** |
| Observations | 16219 | | | | | | | | | | | | | | | 16219 | | 16219 | | 16219 | |

Table S2*:* Model estimates for main and interaction effects (education, gender and age group) for typical occasion quantity

|  | **Main effects** | | | | | | | | | | | | | | | **Education Interaction** | | **Gender Interaction** | | **Age Interaction** | |
| --- | --- | --- | --- | --- | --- | --- | --- | --- | --- | --- | --- | --- | --- | --- | --- | --- | --- | --- | --- | --- | --- |
| *Predictors* | *Estimates* | | | | | | | | | | | | | | *p* | *Estimates* | *p* | *Estimates* | *p* | *Estimates* | *p* |
| (Intercept) | 3.93 | | | | | | | | | | | | | | **<0.001** | 3.83 | **<0.001** | 3.99 | **<0.001** | 4.03 | **<0.001** |
| Age 16-17 (ref) |  |  |  |  |  |  |  |  |  |  |  |  |  |  |  |  |  |  |  |  |  |
| Age 18-19 | 0.15 | | | | | | | | | | | | | | **0.008** | 0.14 | **0.015** | 0.15 | **0.007** | 0.27 | 0.102 |
| Age 20-24 | 0.13 | | | | | | | | | | | | | | **0.009** | 0.12 | **0.012** | 0.13 | **0.007** | 0.26 | 0.082 |
| Age 25-34 | 0.1 | | | | | | | | | | | | | | **0.037** | 0.1 | **0.035** | 0.1 | **0.036** | 0.05 | 0.714 |
| Age 35-44 | 0.01 | | | | | | | | | | | | | | 0.755 | 0.02 | 0.698 | 0.02 | 0.738 | -0.26 | 0.073 |
| Age 45-54 | -0.13 | | | | | | | | | | | | | | **0.008** | -0.12 | **0.009** | -0.12 | **0.009** | -0.26 | 0.077 |
| Age 55-65 | -0.3 | | | | | | | | | | | | | | **<0.001** | -0.3 | **<0.001** | -0.29 | **<0.001** | -0.4 | **0.007** |
| Education Low (up to 10 | 0.16 | | | | | | | | | | | | | | **<0.001** | 0.39 | **<0.001** | 0.16 | **<0.001** | 0.15 | **<0.001** |
| years) |  |  |  |  |  |  |  |  |  |  |  |  |  |  |  |  |  |  |  |  |  |
| Education Mid (11-13 years) | 0.1 | | | | | | | | | | | | | | **<0.001** | 0.24 | **<0.001** | 0.1 | **<0.001** | 0.09 | **<0.001** |
| Education High (13+ years) (ref) | | | | | | |  |  |  |  |  |  |  |  |  |  |  |  |  |  |  |
| Gender [Male] | 0.44 | | | | | | | | | | | | | | **<0.001** | 0.44 | **<0.001** | 0.34 | **<0.001** | 0.43 | **<0.001** |
| Gender [Female] (ref) | | |  |  |  |  |  |  |  |  |  |  |  |  |  |  |  |  |  |  |  |
| IAC Index | -0.03 | | | | | | | | | | | | | | 0.495 | -0.01 | 0.703 | -0.03 | 0.384 | -0.04 | 0.379 |
| Education Low (up to 10 |  | | | | | | | | | | | | | |  | -0.03 | **<0.001** |  |  |  |  |
| years) * IAC Index |  |  |  |  |  |  |  |  |  |  |  |  |  |  |  |  |  |  |  |  |  |
| Education Mid (11-13 years) * IAC Index |  | | | | | | | | | | | | | |  | -0.02 | **0.001** |  |  |  |  |
| Gender [Male] * IAC Index |  | | | | | | | | | | | | | |  |  |  | 0.01 | **0.006** |  |  |
| Age 18-19* IAC Index |  | | | | | | | | | | | | | |  |  |  |  |  | -0.01 | 0.388 |
| Age 20-24* IAC Index |  | | | | | | | | | | | | | |  |  |  |  |  | -0.01 | 0.336 |
| Age 25-34* IAC Index |  | | | | | | | | | | | | | |  |  |  |  |  | 0 | 0.764 |
| Age 35-44* IAC Index |  | | | | | | | | | | | | | |  |  |  |  |  | 0.03 | **0.035** |
| Age 45-54* IAC Index |  | | | | | | | | | | | | | |  |  |  |  |  | 0.01 | 0.315 |
| Age 55-65* IAC Index |  | | | | | | | | | | | | | |  |  |  |  |  | 0.01 | 0.457 |
| Observations | 16169 | | | | | | | | | | | | | | | 16169 | | 16169 | | 16169 | |

Table S3: Model estimates for main and interaction effects (education, gender and age group) for volume of drinking

|  | **Main effects** | | | | | | | | | | | | | | | **Education Interaction** | | **Gender Interaction** | | **Age Interaction** | |
| --- | --- | --- | --- | --- | --- | --- | --- | --- | --- | --- | --- | --- | --- | --- | --- | --- | --- | --- | --- | --- | --- |
| *Predictors* | *Estimates* | | | | | | | | | | | | | | *p* | *Estimates* | *p* | *Estimates* | *p* | *Estimates* | *p* |
| (Intercept) | 7.31 | | | | | | | | | | | | | | **<0.001** | 7.3 | **<0.001** | 7.52 | **<0.001** | 6.96 | **<0.001** |
| Age 16-17 (ref) |  |  |  |  |  |  |  |  |  |  |  |  |  |  |  |  |  |  |  |  |  |
| Age 18-19 | 0.72 | | | | | | | | | | | | | | **<0.001** | 0.67 | **<0.001** | 0.73 | **<0.001** | 1.26 | **0.001** |
| Age 20-24 | 0.9 | | | | | | | | | | | | | | **<0.001** | 0.87 | **<0.001** | 0.91 | **<0.001** | 1.51 | **<0.001** |
| Age 25-34 | 0.92 | | | | | | | | | | | | | | **<0.001** | 0.9 | **<0.001** | 0.92 | **<0.001** | 1.48 | **<0.001** |
| Age 35-44 | 0.88 | | | | | | | | | | | | | | **<0.001** | 0.85 | **<0.001** | 0.88 | **<0.001** | 0.88 | **0.006** |
| Age 45-54 | 0.74 | | | | | | | | | | | | | | **<0.001** | 0.72 | **<0.001** | 0.75 | **<0.001** | 1.08 | **0.001** |
| Age 55-65 | 0.6 | | | | | | | | | | | | | | **<0.001** | 0.58 | **<0.001** | 0.6 | **<0.001** | 1.02 | **0.002** |
| Education Low (up to 10 | 0.15 | | | | | | | | | | | | | | **<0.001** | -0.19 | 0.148 | 0.15 | **<0.001** | 0.15 | **<0.001** |
| years) |  |  |  |  |  |  |  |  |  |  |  |  |  |  |  |  |  |  |  |  |  |
| Education Mid (11-13 years) | -0.01 | | | | | | | | | | | | | | 0.695 | 0.21 | **0.033** | -0.02 | 0.663 | -0.03 | 0.432 |
| Education High (13+ years) (ref) | | | | | | |  |  |  |  |  |  |  |  |  |  |  |  |  |  |  |
| Gender [Male] | 1.14 | | | | | | | | | | | | | | **<0.001** | 1.14 | **<0.001** | 0.78 | **<0.001** | 1.14 | **<0.001** |
| Gender [Female] (ref) | | |  |  |  |  |  |  |  |  |  |  |  |  |  |  |  |  |  |  |  |
| IAC Index | -0.18 | | | | | | | | | | | | | | **0.001** | -0.18 | **0.001** | -0.21 | **<0.001** | -0.14 | **0.02** |
| Education Low (up to 10 |  | | | | | | | | | | | | | |  | 0.04 | **0.006** |  |  |  |  |
| years) * IAC Index |  |  |  |  |  |  |  |  |  |  |  |  |  |  |  |  |  |  |  |  |  |
| Education Mid (11-13 years) * IAC Index |  | | | | | | | | | | | | | |  | -0.03 | **0.017** |  |  |  |  |
| Gender [Male] * IAC Index |  | | | | | | | | | | | | | |  |  |  | 0.04 | **<0.001** |  |  |
| Age 18-19* IAC Index |  | | | | | | | | | | | | | |  |  |  |  |  | -0.06 | 0.114 |
| Age 20-24* IAC Index |  | | | | | | | | | | | | | |  |  |  |  |  | -0.06 | 0.056 |
| Age 25-34* IAC Index |  | | | | | | | | | | | | | |  |  |  |  |  | -0.06 | 0.062 |
| Age 35-44* IAC Index |  | | | | | | | | | | | | | |  |  |  |  |  | 0 | 0.913 |
| Age 45-54* IAC Index |  | | | | | | | | | | | | | |  |  |  |  |  | -0.03 | 0.287 |
| Age 55-65* IAC Index |  | | | | | | | | | | | | | |  |  |  |  |  | -0.05 | 0.171 |
| Observations | 16169 | | | | | | | | | | | | | | | 16169 | | 16169 | | 16169 | |

TableS1: Model estimates for main and interaction effects (education, gender and age group) for frequency of drinking

TableS2: Model estimates for main and interaction effects (education, gender and age group) for typical occasion quantity

TableS3: Model estimates for main and interaction effects (education, gender and age group) for volume of drinking
