# Supplementary material for: Effective alcohol policies are associated with reduced consumption among demographic groups who drink heavily
Source: Alcohol Clin Exp Res (Hoboken). 2023 Apr 23;47(4):786–95. doi: 10.1111/acer.15030 (PMC10947406; doi:10.1111/acer.15030)
Supplement: Supplementary file 2 — Appendix S1 [file ACER-47-786-s001.docx]

**Measures and weighting parameters used for the IAC Alcohol Policy Index**

Countries ranked by IAC Policy Index and domain scores (higher rank and scores indicating more restrictive policy).

|  | Rank | Hours | Outlet density | Drink driving | Pricing | Marketing | Total |
| --- | --- | --- | --- | --- | --- | --- | --- |
| Turkey | 1 | 1.1 | 1.0 | 2.6 | 2.3 | 7.0 | 13.9 |
| Vietnam | 2 | 0.6 | 2.0 | 1.7 | 6.1 | 1.4 | 11.8 |
| Thailand | 3 | 1.3 | 1.0 | 3.0 | 2.2 | 2.8 | 10.3 |
| Mongolia | 4 | 0.7 | 1.0 | 2.1 | 3.2 | 2.7 | 9.7 |
| St Kitts and Nevis | 5 | 1.5 | 2.0 | 0.9 | 1.5 | 2.2 | 8.0 |
| South Africa | 6 | 1.0 | 1.0 | 2.3 | 1.5 | 0.9 | 6.7 |
| Scotland | 6 | 1.1 | 1.5 | 0.4 | 1.9 | 1.6 | 6.5 |
| Australia | 8 | 0.6 | 0.0 | 2.6 | 1.2 | 1.4 | 5.8 |
| England | 9 | 0.7 | 0.0 | 0.9 | 2.3 | 1.3 | 5.1 |
| New Zealand | 10 | 0.4 | 0.0 | 1.8 | 1.5 | 1.4 | 5.0 |
